# Prevalence of HIV, Hepatitis B and C Infections and an Assessment of HCV-Genotypes and Two IL28B SNPs among People Who Inject Drugs in Three Regions of Nepal

**DOI:** 10.1371/journal.pone.0134455

**Published:** 2015-08-11

**Authors:** Hans-Tilmann Kinkel, Dibesh Karmacharya, Jivan Shakya, Sulochana Manandhar, Santosh Panthi, Prajwola Karmacharya, Deepika Sitaula, Reenu Thapaliya, Prawachan K. C., Apurva Rai, Sameer Dixit

**Affiliations:** 1 Gesellschaft für Internationale Zusammenarbeit (GIZ), HRP, PO.Box 1457, Kathmandu, Nepal; 2 Center for Molecular Dynamics Nepal, Kathmandu, Nepal; 3 Trichandra Multiple College, Kathmandu, Nepal; 4 St. Xavier College, Kathmandu, Nepal; 5 SPARSHA Nepal, Latitpur, Nepal; University of Cincinnati College of Medicine, UNITED STATES

## Abstract

As part of a comprehensive health care programme for people who use drugs in Nepal, HIV and viral hepatitis B and C status—including risk factors, HCV-genotypes and co-infections—as well as two IL28B Single-nucleotide polymorphisms (SNPs) were assessed for a random sample of 401 people who inject drugs in three regions of Nepal: mid-western Terrai (Nepalgunj), the eastern region (Dharan, Biratnagar) and the central region (Kathmandu, Lalitpur and Chitwan). Individuals were included who showed at least a minimum of health care seeking behaviour. This latter criterion was defined by being registered with any organisation offering health services. The average age of the participants was 30.5 yrs, and the average length of intravenous drug use was 8.5 yrs. The prevalence rates of HBsAg, anti-HIV antibodies and HCV-RNA were 3.5%, 13.8% and 41.9%, respectively. Spontaneous HCV clearance was evident in 16% of all of those who tested positive for anti-HCV antibodies. Independent risk factors for HCV-RNA positivity were age, gender, geographical region, duration of injecting drug use, history of imprisonment and HIV co-infection. In the age group ≤24 yrs, the rate of spontaneous HCV clearance was 43.5%. Overall, 59.8% of HCV infections were caused by HCV genotype 3 and 40.2% by HCV genotype 1. No other HCV genotypes were identified in this study. The IL28B SNP rs12979860 and rs8099917 were identified in 122 patients, and 75.4% of all participants had both favourable genotypes rs12979860 C/C and rs8099917 T/T.

## Introduction

It has been estimated that 50,000 individuals (approximately 0.3% of the total adult population) inject drugs in Nepal with a significant increase in this figure occurring in recent years [1]. The overwhelming majority of people who inject drugs are young men who inject a pharmaceutical combination of buprenorphine, diazepam and a sedating antihistamine. Heroin use is less common, but does occur [2].

In several urban centres in Nepal, there have been substantial improvements in health care services for people who inject drugs. In particular, opioid substitution therapy, needle and syringe programmes, as well as HIV care and antiretroviral therapy have become more accessible. With these interventions, HIV prevalence has decreased below 10% among people who inject drugs in Nepal [3].

Besides the risk of HIV, people who inject drugs are at high risk of several medical and social harms, such as infection with viral hepatitis B (HBV) and C (HCV), tuberculosis (TB), sexually transmitted infections, mental disorders, and social exclusion. After many years of focussing almost exclusively on combatting HIV, chronic liver disease—and particularly chronic hepatitis C—has become one of the leading causes of mortality for people with a history or current pattern of injecting drug use in many countries [4–7].

HCV is transmitted through blood contact including unsafe injection practices in medical and non-medical (e.g. injecting drug use and tattooing) contexts. Sexual transmission of HCV is relevant among people living with HIV [8]. In 50–80% of cases, HCV can lead to chronic hepatitis and this causes fibrosis, cirrhosis and hepatocellular carcinoma within 10–30 years in up to 30% of patients [9]. In low resource settings, pegylated interferon α plus ribavirin (pegIFN/RBV) is the standard treatment. However, the response to treatment with pegIFN/RBV depends on the viral genotype (HCV-GT) and viral load. In addition to viral factors, host factors also determine the likelihood of achieving a sustained virologic response (SVR) when treating chronic hepatitis C with pegIFN/RBV. Two single-nucleotide polymorphisms (SNP)—IL28B rs12979860 and rs8099917—determine the likelihood of spontaneous viral clearance and SVR with pegIFN/RBV-treatment. Homozygote carriers of cytosine (CC) at position rs12979860, and thymin (TT) at position rs8099917 have a much higher likelihood of spontaneous clearance and achieving SVR with pegIFN/RBV treatment. The higher rate of SVR in Asian patients correlates with high rates of IL28B rs12979860-CC and rs8099917-TT in this part of the world [10–13].

Little is known about HCV prevalence rates in Nepal. While it is low in the general population [14–21], two studies have suggested that the prevalence of anti-HCV antibodies among people with a past and/or current pattern of injecting drug use is around 80% to 85.5% for men and around 15% for women [22–24]. To the best of our knowledge, no systematic assessment of HCV prevalence in Nepal has ever been published with regard to HCV-RNA or genotyping. IL28B-data have been published for several Asian countries [13, 25–30], but there is a lack of published data for Nepal.

Hepatitis B is transmitted vertically, sexually or through blood contact. Chronicity develops in 90% of infants infected with HBV but only 5% of people infected during adulthood. Nepal has the lowest prevalence of hepatitis B in Asia with an HBsAg carrier rate of 0.9% [31]. Apart from a 2011 study on young women, no data on the prevalence of HBV among people who use drugs have been published in Nepal in the last 15 years. In the 2011 study, 13.4% of women had previous exposure to HBV (anti-HBc antibodies) and 0.5% were currently infected (HBsAg) [24].

The main objective of this study was to assess the prevalence rates of HIV, HCV and HBV as well as co- and triple-infection patterns, HCV genotypes and two IL28B-SNPs among a random sample of 401 people who inject drugs and are registered with an organisation providing health care in 3 regions in Nepal. The study was embedded in a comprehensive programme to strengthen healthcare for people who use drugs in Nepal. Its results can be used to tailor a country-specific response to viral hepatitis for people who inject drugs.

## Materials and Methods

In Nepal, all organisations providing health services to people who use drugs are organised under the umbrella group “Recovering Nepal”. The organisations working in the (1) mid-western region Terrai (Nepalgunj), (2) eastern region (Dharan, Biratnagar), and (3) central region (Kathmandu, Lalitpur and Chitwan) were asked to participate in the study. All but one organisation from Kathmandu consented. The organisations gave each patient a code number and then submitted a list of 800 numbers corresponding to their registered clients. Using Microsoft Excel version 2007, a random sample of 480 participants were selected from this list to be included in this cross-sectional/prevalence study. Inclusion criteria were (1) current or past i.v. drug use that fulfils the ICD-10-criteria of opioid dependence, (2) being registered with a “Recovering Nepal”-affiliated organisation and (3) being able and willing to give informed consent. Exclusion criteria were: (1) impossible venepuncture and (2) current or past pharmacological treatment of HCV infection.

Of the 480 initially selected participants, 412 were successfully contacted and enrolled into the study. Eleven of these individuals were subsequently excluded from the analysis because they did not engage in i.v. drug use. As a consequence, 401 participants (301 males, 99 females and 1 transgender who is reported as female based on her own gender definition.) were included in the final analysis. None of the exclusion criteria applied to these randomly selected participants.

The required sample size was calculated for the sub groups, (1) female, (2) male, (3) the eastern region, (4) the mid-west region, (5) Lalitpur and (6) Kathmandu. Lalitpur and Kathmandu are situated in the central valley. Using Raosoft, a sample size of 84 was computed for subgroups 1, 3, 4, 5 and 6 based on an expected HCV prevalence of 15–85%. A desired precision of 10% was given for the prevalence range of 20–80%; the desired precision was 7.5% in the ranges of 15–20% and 80–85%. The required confidence interval was 95% (95% CI).

In confidential one-to-one interviews, trained Nepali counsellors asked participants about their age, gender, length of i.v. drug use as well as their history of sharing injection equipment, history of imprisonment, engagement in sex work and tattooing.

Lab technicians drew 5 ml of venous blood using 5 ml EDTA tubes. This sample was used to determine anti-HIV, anti-HCV, anti-HBs, anti-HBc antibodies, HBsAg, HCV-RNA and HCV-GT.

Assessment of IL28B SNPs investigated the influence of two IL28B SNPs on the natural course of an HCV infection. Participants living with anti-HCV antibodies were asked to provide a second sample of 5 ml EDTA blood once their HCV-status was available; 122 participants were reached and consented to this second step.

An accelerated schedule of HBV-vaccination was offered to all participants who were found to be susceptible to HBV, and all participants living with HIV, HBV or HCV were referred to clinical centres for further assessment. Participants living with HCV were invited to participate in a separate treatment study, which is currently underway.

HIV was diagnosed according to the national standard protocol and employed serial testing, starting with the Determine HIV rapid test (Alere Medical, Japan). In instances with a positive result, this testing was followed by the Unigold HIV rapid test (Trinity Biotech, Ireland). When the results were discordant, the Tridot HIV test (J. Mitra&Co., India) was used as to confirm.

HBV was diagnosed using the Determine HBsAg rapid test (Alere Medical, Japan). This was confirmed by ELISA (Human Diagnostics, Germany). Where results were discordant, the HBV-5 Panel test card from Lumiquick was used as the tie-breaker. Anti-HBc and anti-HBs antibodies were determined with ELISA (Human Diagnostics, Germany). Anti-HCV antibodies were screened using the 4^th^ generation HCV rapid test HCV TRI-DOT (J. Mitra&Co., India) and were confirmed by ELISA (Human Diagnostics, Germany).

The isolation of RNA and RT- PCR was carried out as follows: total RNA was isolated from patients’ serum using the Spin Star viral nucleic acid kit 1.0 (ADT Biotech Sdn Bhd, Malaysia) according to the manufacturer’s instructions. RT-PCR was performed using a two-step RT-PCR. First strand synthesis was performed using the SuperScript II RT-PCR System (Invitrogen, USA) in a 21 μl reaction volume containing 8 μl extracted RNA, 50 ng random hexamers, 1 μl dNTPs mix (10 mM), 9 μl RT buffer and 50 unit superscript II reverse transcriptase. Second strand synthesis was performed in a 25 μl reaction volume using QIAGEN multiplex PCR master mix (# 206143), 0.6 mM sense and antisense primers and 1 μl cDNA. The primers targeting the 5’UTR region as per [32] were forward (DM 50) 5’-CTCGCAAGCACCCTATCAGG-3’ and reverse (DM 51) 5’-GAAAGCGTCTAGCCATGGCGTTAGT-3’. The DNA amplification protocol consists of pre incubation at 95°C for 15 min followed by 40 cycles each of denaturation at 94°C for 15 s, annealing at 55°C for 30 s, and extension at 72°C for 10 min. The last cycle was followed by a 10 min extension at 72°C. The PCR products were analysed by agarose gel electrophoresis. The PCR products of samples showing the expected product size were purified using the ExoSAP-IT PCR product clean up kit (Affymetrix, USA). The purified products were DNA sequenced using the Big Dye Terminator Version 3.1 Cycle Sequencing Kit (Applied Biosystems, USA) with the forward primer DM50. The cycle-sequenced products were purified using the Big Dye X-terminator Kit (Applied Biosystems USA) and analysed in an ABI-3730 genetic analyser (Applied Biosystems, USA). The resulting sequence ends of raw data were trimmed to the reading frame. A BLASTN database search was used to identify the specific genotype.

Phylogenetic Analysis: The HCV isolates were phylogenetically characterized to trace their genetic relatedness with other each other as well as to those from neighbouring countries India, China. Various genotypes of HCV isolates from Thailand as well were used for comparison. A phylogenetic tree was constructed based on their 5UTR region sequence data using MEGA 6.0. A bootstrap re-sampling process (1,000 replications) using the neighbour-joining method was employed to assess the robustness of individual phylogeny nodes.

For IL28B SNP assessment, DNA was extracted from EDTA blood using the DNeasy blood and tissue kit (Qiagen, Germany) according to the manufacturer’s protocol. IL28B SNP genotyping at rs8099917 was carried out using a predesigned TaqMan SNP Genotyping assay (Applied Biosystems, USA) with primers TTGTCACTGTTCCTCCTTTTGTTTT and GGCCCTAACTGATACGCTATAATTAAA. The SNP genotyping at rs12979860 was carried out using a customised TaqMan SNP genotyping assay (Applied Biosystems, USA) that used the primers TGCCTGTCGTGTACTGAACCA and GAGCGCGGAGTGCAATTC as well as the TaqMan probes VIC-TGGTTCGCGCCTTC-MGB and 6FAM-CTGGTTCACGCCTTC-MGB. The RT-PCR was carried out using Primer Design’s lyophilized 2X qPCR mastermix in a 25 μl reaction mix following the manufacturer’s protocol. RT-PCR was carried out with the thermocycling program of one step at 95°C for 15 min followed by 50 cycles of 92°C for 15 s and data collection at 60°C for 1 min.

Excel 7 and SPSS version 22 were used for the statistical analysis of the data. The statistical significance of the analysed frequencies was determined using a χ2-test as well as Fisher’s excact test for any absolute frequency ≤10. Student’s t-test was used to analyse the means of interval variables. Logistic regression was used to measure the relationship between categorical dependent variables and independent variables. Nagelkerke’s R^2^, Hosmer and Lemeshow test as well as the Wald criterion were used to assess the validity of the logistic regression models. P-values <0.05 were considered to be statistically significant.

### Ethics information

The study was divided into three sub-studies—each received separate ethical approval from the National Health Research Council of Nepal (NHRC). The study code 1432 (02 June 2013) included men in all regions, the study code 277 (01 September 2013) included females and the study 888 (29 Jan 2014) referred to the IL28B SNP assessment.

In keeping with the procedures approved by the National Health Research Council (NHRC), all participants were required to give their written consent to participate in the study. The consent and confidentiality declaration was read to each participant in a confidential one-to-one session and was signed (or in case of illiteracy marked with a fingerprint) by all participants. The consent to test for HIV antibodies was obtained separately following pre-test counselling by a counsellor trained according to the national guidelines for HIV counsellors. Participation in the study was also possible for participants who refused the HIV test. Asides from the testing for HIV antibodies, these individuals were included in all information sessions and were subject to all laboratory tests and vaccinations. Participants were given all of their study results in confidential one-to-one counselling sessions.

## Results

The average age of the study participants was 30.5 yrs (median 30 yrs, range 18–56 yrs) with a significant gender difference (females: mean 23.1 yrs, range 18–45 yrs; males: mean 32.9 yrs, range 18–56 yrs, t-test-p<0.001). The mean duration of i.v. drug use for all participants was 8.5 yrs (median 7 yrs, range 0.1–40 yrs) with females having a significantly shorter history of drug use than males (males: 10.3 yrs, females: 3.4 yrs, t-test-p<0.001). The overall life-time history of sharing injection paraphernalia was 62% (males: 69%, females: 40%, χ^2^-p<0.001). When the study population was stratified into groups injecting for ≤5 yrs, 6–9 yrs and ≥10 yrs, the proportion of those reporting a history of sharing injection paraphernalia was 39.8%, 65.8% and 81.8% respectively (χ^2^-p<0.001).

The study revealed that 69% of all participants had a history of imprisonment (males: 84%, females: 23%, χ^2^-p<0.001) with 29% experiencing ≥ 1 month of imprisonment and 8% experiencing ≥ 1 year. Overall, 14% of participants (males: 7.7%, females: 33%, χ^2^-p<0.001) had exchanged sex for money or drugs in the past and 56% (males: 58%, females: 49%, χ^2^-p = 0.11) had intra-cutaneous tattoos or a history of ritual cutting.

### HIV

There were 43 participants who were also registered at “SPARSHA Nepal”, which is an NGO which exclusively serves people living with HIV. Participants from this centre were excluded from the statistical analysis of HIV prevalence to eliminate this obvious selection bias. An additional 4 participants refused an HIV test. Consequently, 354 participants were included in the analysis of HIV prevalence.

After exclusion of the participants from SPARSHA, the overall HIV prevalence in the cohort was 13.8% (95% CI: 10–18%) (Table 1). The differences were not statistically significant—females had lower rates than males, and those living in the mid-western Nepalguni region had lower rates than those in the central and eastern regions.

**Table 1:** HIV infection stratified by gender and region of Nepal.

	All			Eastern Region			Central Region			Nepalgunj		
M/F	All	M	F	All	M	F	All	M	F	All	M	F
**n**	354	254	100	139	70	69	136	108	28	79	76	3
**%**	100%	72%	28%	39%	20%	19%	38%	31%	8%	22%	21%	0.9%
**HIV**	49	40	9	21	12	9	22	22	0	6	6	0
**No HIV**	305	214	91	118	58	60	114	86	28	73	70	3
**HIV (%)**	**13.8%**	**15.7%**	**9.0%**	**15.1%**	**17.1%**	**13.0%**	**16.2%**	**20.4%**	**0.0%**	**7.6%**	**7.9%**	**0.0%**
**95% CI**	10–18%	12–21%	5–16%	10–22%	10–28%	7–23%	11–23%	14–29%	0–12%	4–16%	4–16%	0–56%

M: male, F: female, 95% CI: 95%-confidence interval. n = 354. Gender difference (male vs. female) for HIV non-significant, χ^2^-p = 0.18. Regional difference (Nepalgunj vs. ‘other’) for HIV non- significant χ^2^-p = 0.07.

Besides other factors (region and history of sharing injecting paraphernalia), the duration of injecting drug use (in years) was the strongest significant predictor of the presence of anti-HIV antibodies in a logistic regression model (Exp(B) 1,215, p<0.001). When stratified into groups of people injecting for ≤5 yrs, 6–9 yrs and ≥10 yrs, the prevalence of anti-HIV antibodies was 3.6%, 13.9% and 43.6%, respectively (χ^2^-p<0.001).

### HBV

Lifetime exposure to HBV (anti-HBc antibodies) was found in 43.8% of the study population (95% CI: 39–49%). Besides other factors (age and region), the duration of injecting drug use (in years) was the strongest significant predictor of presence of anti-HBc antibodies in a logistic regression model (Exp(B) 1,178, p<0.001). When stratified in groups of people injecting for ≤5 yrs, 6–9 yrs and ≥10 yrs, the prevalence of anti-HBc antibodies was 22.4%, 43.8% and 65.4%, respectively (χ^2^-p<0.001). The overall HBV prevalence (HBsAg positive) was 3.5% (95% CI: 2–6%) (Table 2) with a non-significant trend (p = 0.07) towards higher rates in the Terai regions (Biratnagar and Dharan in the eastern region and Nepalgunj in western region) versus the central region (Lalitpur and Kathmandu). Of the 401 participants, 78 (19.5%) had anti-HBs antibodies as markers of naturally- or vaccination-acquired immunity.

**Table 2:** HBV infection stratified by gender and region of Nepal.

	All			Eastern Region			Central Region			Nepalgunj		
M/F	All	M	F	All	M	F	All	M	F	All	M	F
**n**	401	301	100	141	72	69	181	153	28	79	76	3
**%**	100%	75%	25%	35%	18%	17%	45%	38%	7%	20%	19%	0.75%
**HBsAg**	14	10	4	8	5	3	3	2	1	3	3	0
**No HBsAg**	387	291	96	133	67	66	178	151	27	76	73	3
**HBsAg (%)**	**3.5%**	**3.3%**	**4%**	**5.7%**	**6.9%**	**4.3%**	**1.7%**	**1.3%**	**3.6%**	**3.8%**	**3.9%**	**0.0%**
**95% CI**	2–6%	1.7–6.2%	1.5–10%									

M: male, F: female, HBsAg: Hepatitis B surface antigen, 95% CI: 95%-confidence interval, n = 401. Gender difference (male vs. female) for HBsAg non-significant, χ^2^-p = 0.75. Regional difference (Central vs. ‘other’) for HBsAg non-significant χ^2^-p = 0.07.

A total of 31% of all HBsAg-negative participants showed an “isolated anti-HBc antibodies” pattern (i.e. anti-HBc antibody positive and HBsAg/anti-HBs antibody negative), and 6.3% of all participants tested positive for isolated anti-HBs antibodies after vaccination. Of the 26 people who stated having been vaccinated against HBV in the past, the following results were observed: 2 (7.7%) were HBsAg postive, 4 (15.4%) were isolated anti-HBc antibody carriers, 6 (23.1%) were naturally immune (anti-HBc and anti-HBs antibodies), and 7 (27%) had neither anti-HBc nor anti-HBs antibodies. Seven individuals (27%) had isolated anti-HBs antibodies. Details about HBV/HCV co-infection are discussed in the section below.

Antiretroviral treatment with 3TC and/or TDF is the standard first line therapy for HIV in Nepal. 3TC and/or TDF are induce HBs-seroconversion within 7 years in up to 10% of people with chronic HBV-infection [33]. However, we did not assess levels of ART use for the study participants. Among all participants with anti-HBc antibodies and known HIV status, HBsAg was present in 5 of the 70 HIV-positive (7.1%) participants and 9 of the 103 HIV-negative (8.7%) participants (Fisher’s exact-p = 0.78).

### HCV

The overall prevalence of anti-HCV antibodies in the tested population was 49.9%. Besides other factors (age, region, history of imprisonment and history of sharing injection paraphernalia) the duration of i.v. drug use (in years) was the strongest significant predictor for the presence of anti-HCV antibodies in a logistic regression (Exp(B) 1,326, p<0.001). When stratified into groups of people injecting for ≤5 yrs, 6–9 yrs and ≥10 yrs, prevalence of anti-HCV antibodies was 19.3%, 46.6% and 84.3%, respectively (χ^2^-p<0.001).

**Fig 1:**
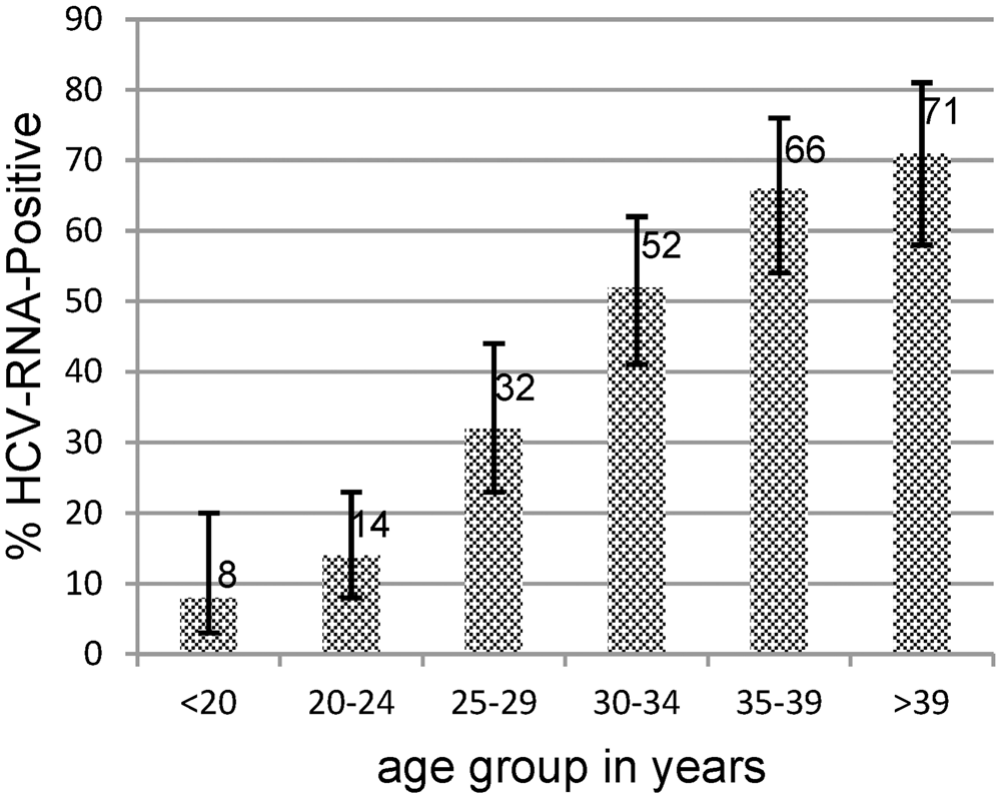
HCV-RNA positivity stratified by age. Rate of HCV-infection is associated with age (χ^2^-p<0.001, error bars show 95% CI).

The prevalence of HCV-RNA was 41.9% (95% CI: 37–47%) with significantly higher rates in the central and eastern regions than in the western region (Nepalgunj) (49.7% vs 16.5%, χ^2^-p<0.01) (Table 3). HCV-RNA prevalence correlated significantly with age (Fig 1). A logistic regression analysis was used to analyse the factors associated with HCV infection and to predict the HCV-RNA positivity. Independent variables included (1) history of imprisonment, (2) duration of injecting drug use, (3) age, (4) region, (5) gender and (6) HIV status (Table 4).

**Table 3:** HCV infection stratified by gender and region of Nepal.

	All			Eastern Region			Central Region			Nepalgunj		
M/F	All	M	F	All	M	F	All	M	F	All	M	F
**n**	401	301	100	141	72	69	181	153	28	79	76	3
**%**	100%	75%	25%	35%	18%	17%	45%	38%	7%	20%	19%	0.75%
**HCV naive**	201	120	81	74	22	52	66	40	26	61	58	3
**Anti-HCV-Ab**	200	181	19	67	50	17	115	113	2	18	18	0
**HCV-RNA**	168	159	9	51	44	7	104	102	2	13	13	0
**HCV-RNA(%)**	**41.9%**	**52.8%**	**9.0%**	**36.2%**	**61.1%**	**10.1%**	**57.5%**	**66.7%**	**7.1%**	**16.5%**	**17.1%**	**0%**
**95% CI**	37–47%	47–58%	5–16%									

M: male, F: female, AB: antibodies, RNA: ribonucleic acid, 95% CI: 95%-confidence interval, n = 401. Gender difference (male vs. female) for anti-HCV antibodies and HCV-RNA significant, both: χ^2^-p<0.01, but the difference is not evident once gender is adjusted for age (see main text). Regional difference (Nepalgunj vs. ‘other’) for anti-HCV antibodies and HCV-RNA both: significant χ^2^-p<0.01.

**Table 4:** Logistic regression model to predict HCV-RNA positivity.

	B	S.E.	Wald	df	Sig.	Exp(B)	95% CI for EXP(B) Lower	95% CI for EXP(B) Upper
**Region**			32.166	2	.000			
**Region(1)**	3.142	.564	31.009	1	.000	23.150	7.661	69.955
**Region(2)**	2.464	.500	24.234	1	.000	11.750	4.406	31.337
**Age**	.084	.027	9.857	1	.002	1.088	1.032	1.146
**started**	.150	.037	16.473	1	.000	1.162	1.081	1.250
**everprison(1)**	1.563	.492	10.092	1	.001	4.772	1.820	12.514
**HIV(1)**	2.764	.578	22.853	1	.000	15.864	5.108	49.270
**Gender**	-1.968	.665	8.771	1	.003	.140	.038	.514
**Constant**	-5.997	1.468	16.695	1	.000	.002		

Variables encoded as:

Have you ever been in **prison**? (Yes = 1; No = 0)

How many years ago have you **started** injecting drugs? In years

What is your **age**? In years

In which **region** does the person live? (Region 0: Western (Yes = 1; No = 0); Region 1: Eastern Yes = 1;No = 0); Region 2: Central (Yes = 1; No = 0),

Is the person living with **HIV**? (Yes = 1; No = 0)

Gender? (Female: 1; Male = 0)

A test of the full model against a constant-only model was statistically significant indicating that the predictors reliably distinguished between those who were living with HCV and those who were not (χ^2^ = 277.7, p<0.001 with df = 7). A Nagelkerke’s R^2^ of 0.69 indicated that there was a moderately strong relationship between prediction and grouping. A Hosmer and Lemeshow test produced a p = 0.775, indicating that the model provided a good fit to the data. The Wald criterion demonstrated that all parameters made a significant contribution to the model prediction (p≤0.001). Seven “outliers” were removed from the model. The prediction formula was given as:

$$\text{p}=\frac{{\text{e}}^{\left(-5.997+0.15\times \text{Started}+1.563\times \text{Prison}+0.084\times \text{Age}+3.142\times \text{East}+2,464\times \text{Central}+1\times \text{Western}+2.764\times \text{HIV}-1.968\times \text{Female}\right)}}{1+{\text{e}}^{\left(-5.997+0.15\times \text{Started}+1.563\times \text{Prison}+0.084\times \text{Age}+3.142\times \text{East}+2,464\times \text{Central}+1\times \text{Western}+2.764\times \text{HIV}-1.968\times \text{Female}\right)}}$$

Prediction success was 86.5% (91.2% for HCV-negativity and 80% for HCV-positivity) with an overall cut-off value of p = 0.5.

### Persistence and spontaneous clearance of HCV

**Fig 2:**
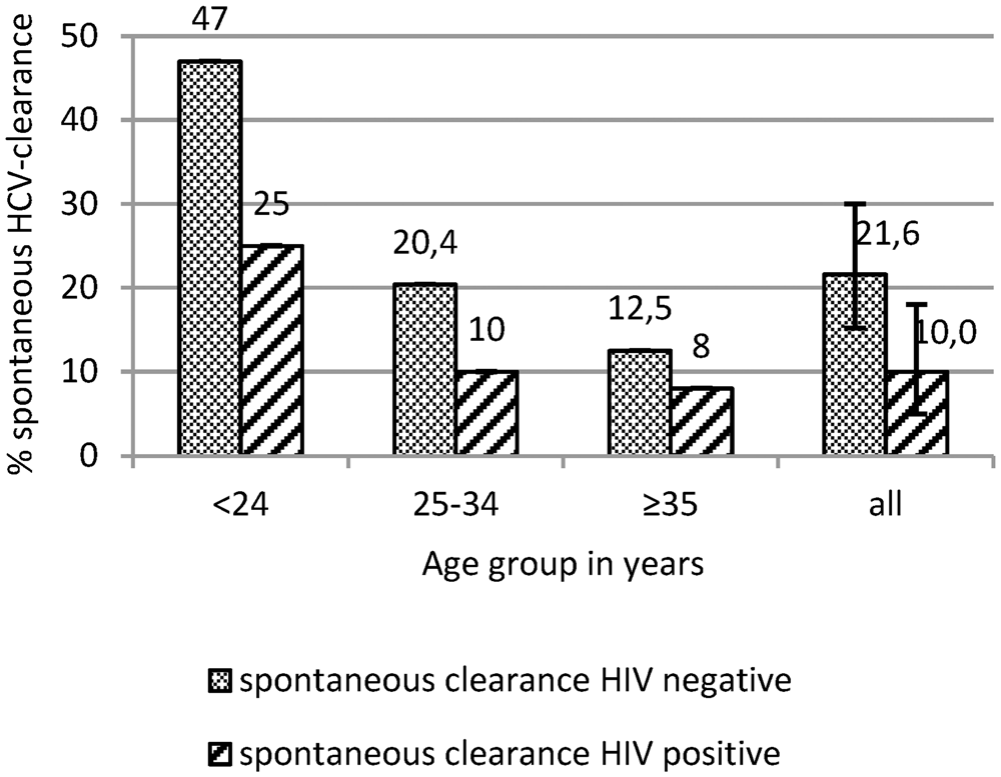
Rate of spontaneous clearance stratified by age and HIV status. Rate of spontaneous clearance of HCV is associated with age (spontaneous clearance associated with age: χ^2^-p<0.001) and HIV status (spontaneous clearance associated with HIV-status in “all” patients: two-sided Fischer’s Exact p = 0.03, error bars show 95% CI).

Of the 200 patients who were reactive for anti-HCV antibodies, 32 (16%, 95% CI: 12–22%) experienced spontaneous HCV-clearance (anti-HCV positive and HCV-RNA negative). This spontaneous clearance occurred in 43.5% of those ≤24 yrs (Fig 2). In a logistic regression model for all participants with anti-HCV antibodies, the persistence of HCV-RNA correlated significantly with age (p = 0.01, Exp(B) = 1.071, 95% CI: 1.016–1.128).

The spontaneous HCV clearance rate was significantly lower in people living with HIV (people living without HIV: 21.6%, 95% CI: 15–30%; people living with HIV: 10%, 95% CI: 5–18%, Fisher’s exact-p = 0.025) (Fig 2). In our study, the gender differences in spontaneous HCV clearance disappeared when adjusting for age.

### HCV genotypes

**Fig 3:**
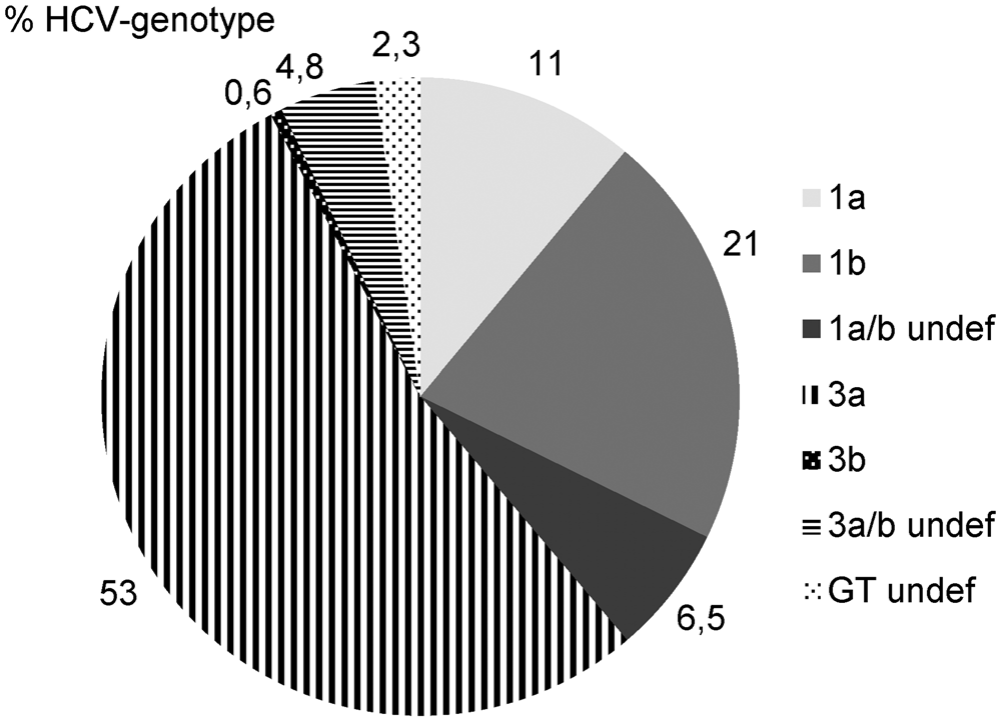
Percentage of HCV genotype-frequencies.

**Fig 4:**
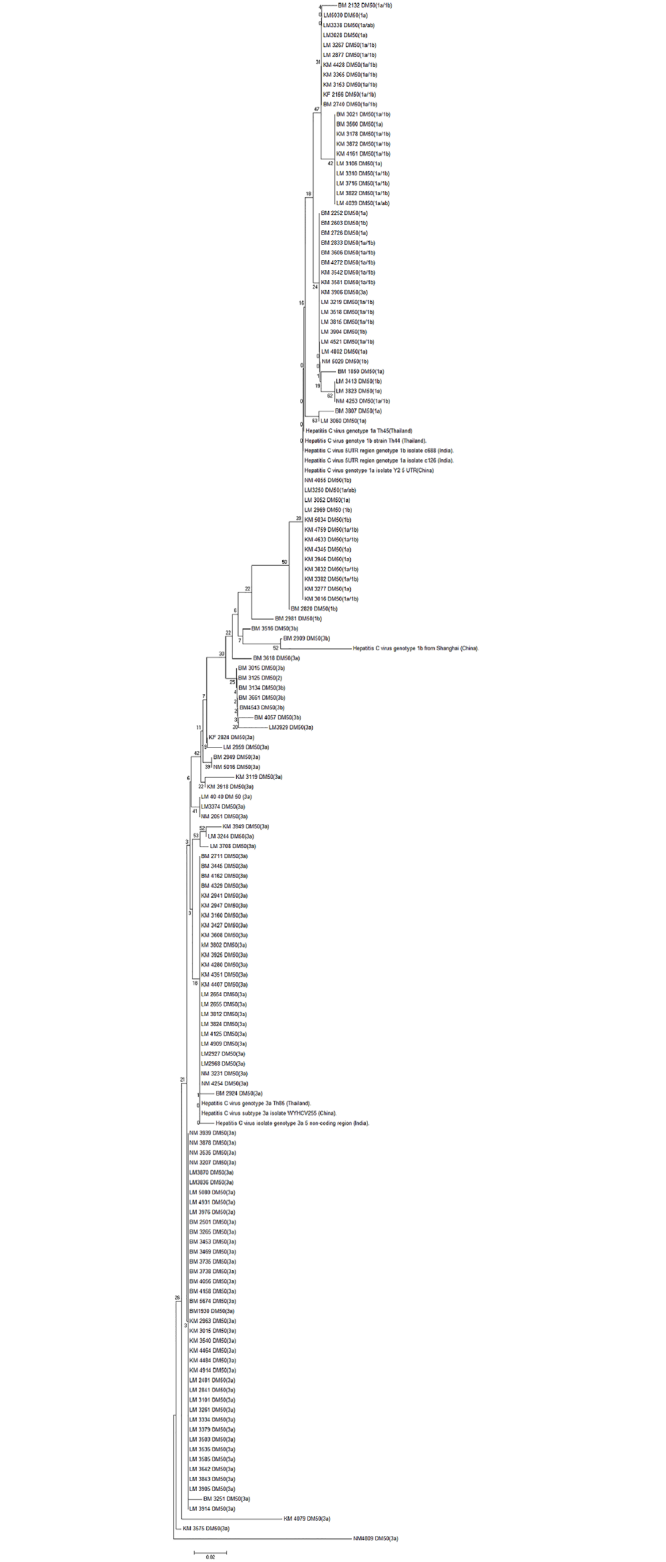
Phylogenetic tree of the 5 UTR region of HCV. The tree is generated by the neighbour-joining method in MEGA 6. The symbol indicates the reference sequences. The first character of the code indicates the origin of the sample (K: Kathmandu, L: Lalitpur, (Central Region), B: Biratnagar (Eastern Region), and N: Nepalgunj (Western Region).

HCV genotypes(HCV-GT) were determined in 164 of the 168 HCV-RNA positive participants and HCV-GT remained undetermined in 4 samples. Of the 164 samples with a determined HCV-GT, 66 were HCV-GT 1 (40.2%), and 98 were HCV-GT 3 (59.8%) (Fig 3). There was no significant difference in HCV-GT in terms of gender or region (Table 5). Subtype 3b was found exclusively in the eastern region where 25.8% of all HCV-GT3 infections were caused by subtype 3b, and the remaining 74.2% were caused by subtype 3a. All HCV-GT 3 viruses were subtype 3a in the other two regions and one undefined GT3 subtype was found in the central region. The phylogenetic tree of the 5UTR region (Fig 4) of the HCV isolates showed a genotype-specific clustering within samples and with reference samples from isolates of India, China and Thailand.

**Table 5:** HCV-genotypes stratified by gender and region.

	All			Eastern Region			Central Region			Nepalgunj		
M/F	All	M	F	All	M	F	All	M	F	All	M	F
**n**	168	159	9	58	44	14	104	102	2	12	12	0
**%**	100%	91%	9.0%	33%	25%	8%	60%	59%	1.1%	6,9%	6,9%	0%
**GT1**	**66**	**61**	**5**	**20**	**16**	**4**	**43**	**42**	**1**	**3**	**3**	**0**
**GT1a**	19	18	1	7	6	1	12	12	0	0	0	0
**GT1b**	11	10	1	4	3	1	5	5	0	2	2	0
**GT1a/b undef**	36	33	3	9	7	2	24	24	1	1	1	0
**GT3**	**98**	**94**	**4**	**31**	**28**	**3**	**57**	**56**	**1**	**10**	**10**	**0**
**GT3a**	89	85	4	23	20	3	56	55	1	10	10	0
**GT3b**	8	8	0	8	8	0	0	0	0	0	0	0
**GT3a/b undef**	1	1	0	0	0	0	1	1	0	0	0	0
**GTundef**	4	4	0	0	0	0	4	4	0	0	0	0

M: male, F: female, “undef” = undefined, n = 200. Gender difference for GT1 vs. 3 non-significant χ^2^-p = 0.34. Regional difference for GT1 vs. 3 non-significant χ^2^-p = 0.38.

### HBV/HCV co-infection and HBV/HCV/HIV triple infection

Only 3 of the 401 participants were found to have an HBsAg-positive HBV infection and an RNA-positive HCV infection on the day of examination (0.75%). Two of these patients were also living with HIV (“triple infection”).

**Fig 5:**
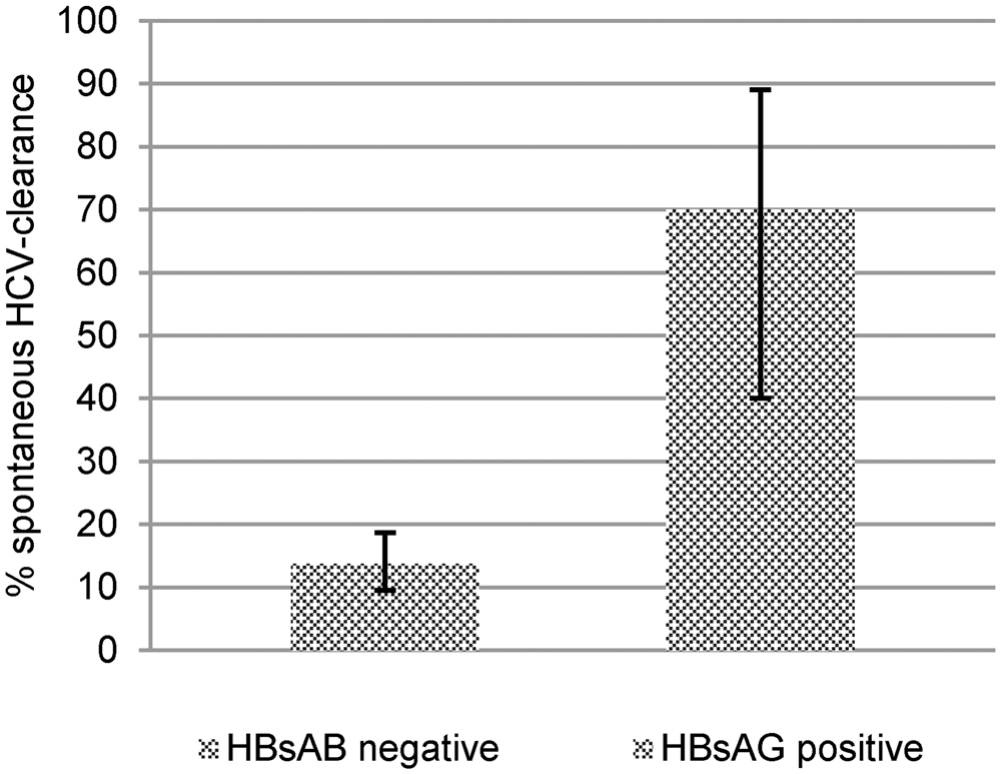
Rate of spontaneous clearance stratified by HBsAg-status. Spontaneous HCV clearance among people living with HBsAg is significantly higher versus people who are negative for HBsAg. (Two-sided Fisher exact p<0.001, error bars show 95% CI).

Of all participants who were found to be reactive for anti-HCV antibodies, 70% of those living with HBV (HBsAg positive) experienced spontaneous HCV clearance, while only 13.7% of HBsAG-negative participants experienced a spontaneous HCV clearance. (Fisher’s exact-p<0.001) (Fig 5). However, HBV-status was not statistically significantly associated with the very high rates of spontaneous HCV-clearance in the age group ≤24 yrs.

When only people with both anti-HBc antibodies and anti-HCV antibodies are analysed (n = 140), the “isolated anti-HBc”-pattern was found in 71.6% of people with HCV-persistence and 41.7% with spontaneous HCV clearance (Fisher’s exact-p = 0.008). HBsAg was diagnosed in 29.2% of people with spontaneous HCV clearance and 2.6% of people with HCV-persistence (Fisher’s exact-p<0.001) (Table 6)

**Table 6:** HCV/HBV co-infection.

	HBV: naturally immune (anti-HBs- and anti-HBc)	HBV: isolated anti-HBc	HBsAG positive
**HCV-RNA positive (n = 116)**	30 (25.9%)	83 (71.6%)	3 (2.6%)
**HCV-RNA negative (n = 24)**	7 (29.2%)	10 (41.7%)	7 (29.2%)

All participants with lifetime markers of previous infection of HBV (anti-HBc antibodies) and HCV (anti-HCV antibodies) (n = 140). Isolated-anti-HBc pattern is significantly more frequent in HCV-viraemic participants (Fisher’s exact p = 0.008); HBsAg prevalence is significantly more frequent in people with spontaneous HCV-clearance (Fisher’s exact p<0.001).

### HIV/HCV co-infection

**Fig 6:**
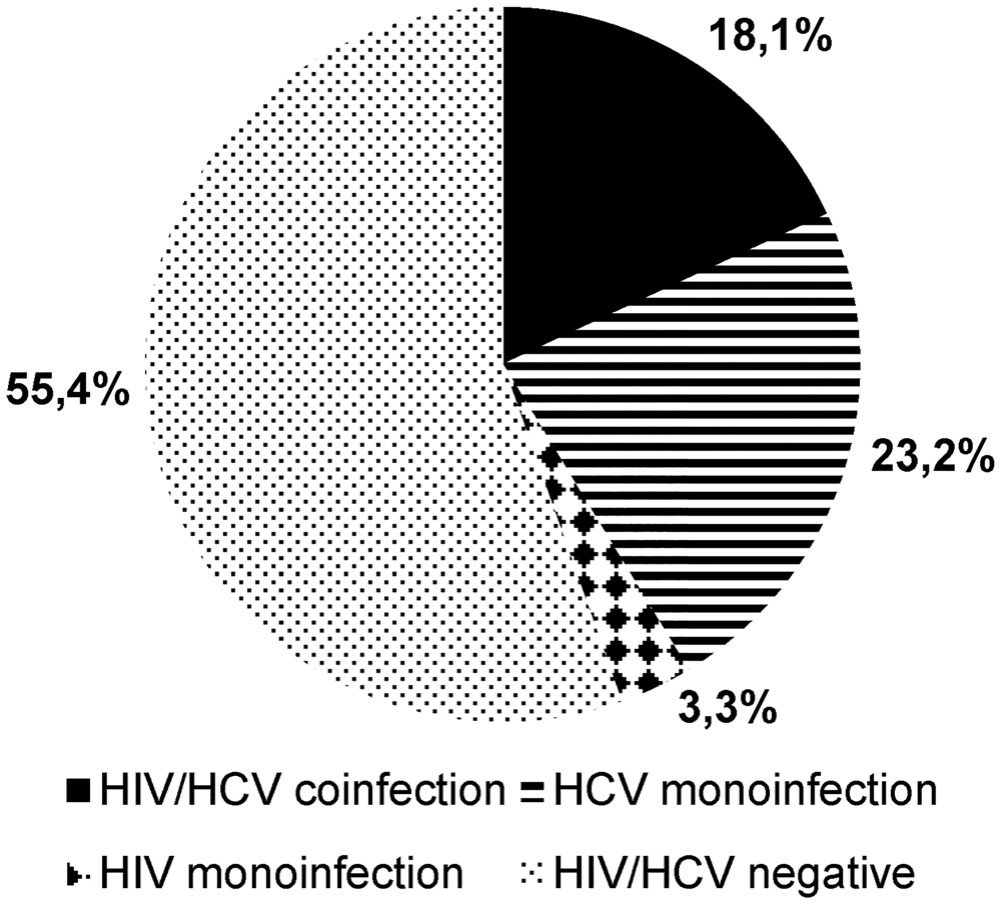
Percentage of frequencies of HIV and HCV mono- and co-infection. At 3.3%, HIV mono-infection is a comparatively rare constellation whereas HIV/HCV co-infection is found in 18.1% of all participants (n = 397).

Of the 397 participants with known HIV-status, 72 (18.1%) were living with an HIV/HCV co-infection (HCV-RNA and anti-HIV-antibodies) (Fig 6). Participants living with HIV had an HCV-RNA-positivity rate of 84.7%, whilst HIV-negative participants showed an HCV-RNA-positivity rate of 29.5% (χ^2^-p<0.001).

### IL28B

IL28B SNPs rs12979860 and rs8099917 were determined in 122 participants—20 were anti-HCV antibody reactive and HCV-RNA negative (“spontaneous clearance”) and 102 were HCV-RNA positive (Table 7). IL28B rs8099917 T/T is associated with a higher rate of spontaneous clearance—all 20 patients with spontaneous clearance (anti-HCV antibody reactive, HCV-RNA negative) had rs8099917 T/T whilst 20 out of 102 (19.7%) patients currently living with HCV (anti-HCV-antibody reactive, HCV-RNA positive) had IL28B rs8099917 G/T (Fisher’s exact-p = 0.04). Participants for the IL28B assessment were selected to examine its effect on the natural course of the HCV-infection. However, the 30 participants with heterozygote IL28B alleles in one or both IL28B SNPs also had a non-significant trend towards impaired clearance of HBsAg versus carriers of IL28B rs12979860-CC/rs8099917-T/T (Fisher’s exact-p = 0.09).

**Table 7:** I28B SNP rs12979860 and rs8099917.

IL28B	rs8099917 T/T	rs8099917 G/T	rs8099917 G/G
**rs12979860 C/C**	92	1	0
% (95% CI)	75.4% (67–82%)	0.8% (0.1–4.5%)	0%
**rs12979860 C/T**	10	18	0
% (95% CI)	8.2% (4.5–14%)	15% (10.5–22%)	0%
**rs12979860 T/T**	0	1	0
% (95% CI)	0%	0.8% (0.1–4.5%)	0%

N = 122

## Discussion

This study is a baseline assessment to tailor a country-specific response to viral hepatitis among people who inject drugs in Nepal. The results detail the current infection rates among people who inject drugs and seek health care; they do not constitute a nationally representative prevalence survey. Versus national surveillance data (CBS-studies) among people who use drugs, the average age of the participants in our study (30.5 yrs) is higher than the average age of all people who inject drugs in Nepal. The average duration of i.v. drug use (8.5 yrs) is also longer than the average age of those interviewed for the CBS studies (CBS average age is 25.07 yrs with 66% reporting drug use for ≤5 yrs) [1]. The prevalence rates of HBsAg, HIV and anti-HCV antibodies were 3.5%, 13.8% and 49.9%. These figures are 4-, 50- and 100-times higher than the reported national averages. The national figures are mostly based on data from blood donation centres and the “2011-Integrated Biological and Behavioural Surveillance” (IBBS) HIV study [3]. The latter was based on a respondent-driven sample in Pokhara (western region) and Kathmandu. In contrast to our study, this data suggested that the prevalence of HIV among people who inject drugs was as low as 4.6% and 6.3% [3].

In comparison to previous studies [22, 23], our estimates for 2013 suggest that the prevalence of anti-HCV antibodies and HCV-RNA is lower—only 49.9% and 41.9%, respectively. The lower rates of anti-HCV antibodies in our sample could have several explanations: (1) females accounted for 25% of our study participants whereas the female/male ratio among people who inject drugs in Nepal is usually much lower (e.g. <5–8%) in respondent-driven samples [1, 3]. In our study, HCV prevalence among women who use drugs was also much lower than among men, which is similar to results from an UNODC study from 2011 that found that 15.2% of women who inject drugs were anti-HCV antibody positive [24]. (2) The lower HCV prevalence found might also reflect a reduction in HCV incidence resulting from the establishment of harm-reduction services. Sharing needles with others was reported by only 5% of participants in 2011, whereas the comparable figures for the Kathmandu valley in 2002 and 2005 were 59% and 27%, respectively [3]. The idea that there has been a reduction in HCV incidence is supported by the fact that 80.7% of people in our study who had injected drugs for ≤ 5 yrs had never contracted HCV, whereas 84.3% of those who had injected for ≥ 10 yrs had anti-HCV antibodies. Previous assessments found that without preventive interventions, HCV infection usually occurs very quickly after initiation of i.v. drug use [34, 35]. The low rate of anti-HCV antibodies among people with a history of injecting drugs for ≤5 yrs is paralleled by similar reductions in individuals’ history of sharing injection paraphernalia as well as lower rates of anti-HIV and anti-HBc antibodies. (3) A persistently high level of mortality is another factor that could account for the comparatively low prevalence of HCV in our study. In 2011, there were 4,722 HIV-related deaths among people living with HIV—this constitutes 9.4% of the overall number of people living with HIV in Nepal. This is one of the highest proportions of any country in the world [36, 37].

Here, we defined a logistic regression formula that enables us to discriminate between those with the highest and lowest pre-test probability of HCV where HCV-laboratory testing is not readily available.

Almost 50% of people ≤ 24 yrs who are living with anti-HCV achieved spontaneous clearance (defined as anti-HCV antibody positive but HCV-RNA negative). This rate is higher than the previously reported rates for most other settings or ethnicities [38, 39]. The high rate of spontaneous clearance could be explained at least in part by the favourable IL28B SNPs [40]. We found favourable IL28B SNP rs12979860 and rs8099917 in 76.2% and 83.6% of participants; 75% of participants had favourable variants in both IL28B SNPs. These findings are in line with Firdaus et al.’s study of different population groups in eastern and north-eastern parts of India [30]. We found a significant correlation between IL28B rs8099917 T/T and spontaneous clearance. Because homozygote IL28B rs12979860-C/C and -rs8099917-T/T are associated with high SVR rates after treatment with pegIFN/RBV, our data supports increased availability of HCV-treatment programs [10–13, 30].

The 60:40% ratio of HCV-GT3/GT1 among people with RNA-positive HCV infection is also in line with the findings of Firdaus et al. In their work, they reported a 66:32% distribution from eastern and north-eastern India [35]. The genotype-specific clustering of the HCV isolates indicates absolute genetic relatedness regardless of geographic origin.

Hepatitis B is a relatively rare infection in Nepal versus the neighbouring countries India and China [41, 42]. The overall prevalence in the general population has repeatedly been found to be <2% [43]. This pattern is confirmed by our findings where even in a high-risk population, the prevalence of HBsAg was just 3.3%. However, we found a high rate of anti-HBc antibodies (49.1%) in our cohort, which illustrates an increased risk of infection. It is possible that this mainly occurs during adolescence and adulthood and leads to an acute hepatitis B with subsequent HBsAG clearance. However, our findings also support previous reports that HBV and HCV can, to a certain degree, suppress each other’s replication [34, 40]. Furthermore the high rate of “isolated anti-HBc” patterns especially among people living with HIV or HCV might indicate that there is a relevant proportion of occult HBV infection [44].

HCV/HIV co-infection is common, and 18% of all participants in the study were found to be HIV/HCV co-infected. HIV accelerates the progress of HCV-induced liver disease [9] and putting HIV/HCV co-infected patients on effective ART seems to lessen this effect [45, 46]. While the WHO currently states that no general recommendation can be given to start ART earlier in patients living with HCV/HIV co-infection [47], it is important to include HCV care into the spectrum of services for people living with HIV. This is especially true in Nepal: 85% of all people who inject drugs and live with HIV were co-infected with HCV, and only 13 (3.3%) of the 397 HIV-tested participants presented with an HIV mono-infection.

In conclusion, the study shows that the potential HCV treatment needs for people who inject drugs in Nepal are lower in absolute numbers than anticipated. Furthermore, we infer that it is reasonable to expect high SVR rates for treatment with pegIFN/RBV because most patients living with HCV are infected with GT3 and have a combination of favourable IL28B SNPs. Finally, we conclude that HCV/HIV co-infection is frequent in Nepal whereas HIV mono-infection is rare. As such, all people living with HIV should be screened, diagnosed and evaluated for HCV therapy. Clinicians should consider initiating ART shortly after infection.

### Limitations

The study sample does not constitute a representative sample of all people who use or inject drugs in Nepal. The study only covers individuals living in Dharan, Biratnagar, Kathmandu, Lalitpur, Chitwan, and Nepalgunj. However, there are also other places with non-ignorable numbers of people who use drugs (Pokhara, Budwal, Birganj and the far-western region). The sample is also not representative for all people who inject drugs in the regions where our study was conducted. In particular, the study sample over-represents females and only includes people with health-care seeking behaviour. As a consequence, the study tends to include older participants with a comparatively longer duration of i.v. drug use. It is likely that our HIV-prevalence estimate is actually rather conservative because we excluded participants registered with SPARSHA for the estimation of HIV-prevalence. This is especially true when considering HIV-prevalence in the central valley (Kathmandu and Lalitpur) where people living with HIV are mainly concentrated at SPARSHA.

The technique that was used to sequence a 150 bp sequence of the 5’UTR region of the viral genome failed to define HCV GT in 4 of the 168 HCV-RNA positive samples.
